# Reoperative Pulmonary Valve Replacement via Left Mini-Thoracotomy in a Young Woman With Absent Pulmonary Valve Syndrome: A Case Report

**DOI:** 10.7759/cureus.107900

**Published:** 2026-04-28

**Authors:** Luis A Morales-Díaz, Adan Becerril Ponce, Hector F Ortiz-Almeyda, Jonhatan M Cota-Arce, luis Manuel Alvárez Sánchez

**Affiliations:** 1 Cardiothoracic Surgery, Instituto Mexicano del Seguro Social, Mexico City, MEX; 2 Cardiology, Instituto Mexicano del Seguro Social, Mexico City, MEX

**Keywords:** absent pulmonary valve, bioprosthetic valve, congenital heart disease, left mini thoracotomy, pulmonary artery dilation, pulmonary valve replacement, reoperative cardiac surgery, right ventricular outflow tract, structural valve degeneration, thoracotomy approach

## Abstract

Absent pulmonary valve syndrome is a rare congenital cardiac anomaly characterized by severe pulmonary valve insufficiency and progressive dilation of the right ventricular outflow tract and pulmonary arteries. Although primary surgical repair is typically performed during childhood, long-term outcomes are frequently limited by structural degeneration of bioprosthetic valves, often leading to the need for reintervention in early adulthood.

We present the case of a 22-year-old woman with a history of pulmonary valve agenesis who underwent surgical correction during childhood, including implantation of an Edwards N21 bioprosthetic pulmonary valve and pulmonary artery plication. During follow-up, she developed progressive exertional dyspnea associated with a decline in functional capacity and was in New York Heart Association functional class III at the time of reoperation. Imaging studies demonstrated structural deterioration of the prosthetic valve, with evidence of right ventricular outflow tract involvement and hemodynamic compromise, supporting the indication for surgical reintervention.

Pulmonary valve re-replacement was successfully performed through a left mini-thoracotomy approach, allowing adequate exposure of the right ventricular outflow tract and main pulmonary artery while avoiding the risks associated with repeat sternotomy. The degenerated prosthesis was explanted and replaced with a new 25 mm Edwards bioprosthetic pulmonary valve, achieving satisfactory intraoperative hemodynamics and valve function. The postoperative course was uneventful, and the patient was discharged with significant clinical improvement and recovery of functional status.

This case highlights that a left mini-thoracotomy approach represents a feasible and safe alternative for pulmonary valve re-replacement in selected patients with prior sternotomy. In addition to providing effective surgical exposure, this strategy may reduce operative trauma and facilitate postoperative recovery in complex reoperative congenital cardiac cases.

## Introduction

Absent pulmonary valve syndrome is an uncommon congenital cardiac anomaly characterized by hypoplastic or absent pulmonary valve leaflets, resulting in severe pulmonary regurgitation and progressive dilation of the pulmonary arteries and right ventricular outflow tract. It is most frequently associated with tetralogy of Fallot, although isolated variants have also been described [1-4]. The clinical course is often determined early in life, as significant airway compression and right ventricular volume overload may necessitate surgical intervention during infancy or childhood.

Although surgical management has evolved and early survival has improved, long-term outcomes remain influenced by progressive right ventricular remodeling and the durability of valve substitutes. Bioprosthetic valves are commonly used in the pulmonary position due to their favorable hemodynamic profile and the avoidance of long-term anticoagulation. However, structural valve degeneration remains an expected limitation, particularly in younger patients, frequently leading to the need for reintervention [5-8].

Reoperative cardiac surgery after prior sternotomy is associated with increased technical complexity and perioperative risk, largely due to mediastinal adhesions and the potential for injury during re-entry. In this context, alternative surgical strategies have been explored to mitigate these risks. Minimally invasive approaches, including left mini-thoracotomy, may provide adequate exposure of the right ventricular outflow tract and pulmonary artery while avoiding repeat sternotomy in selected patients. In our case, the left mini-thoracotomy was performed through the fourth left intercostal space, which provided adequate exposure for pulmonary valve re-replacement. The procedure was performed under general anesthesia with orotracheal intubation and invasive hemodynamic monitoring. After systemic heparinization and confirmation of adequate activated clotting time, cardiopulmonary bypass was established through peripheral femoral arterial and venous cannulation. Pain control was achieved using a multimodal analgesic strategy, including regional anesthesia, local anesthetic infiltration of the thoracotomy wound, and opioid rescue as needed. Recent reports have demonstrated the feasibility and safety of these approaches in patients undergoing pulmonary valve replacement, with favorable early and mid-term outcomes [9-12]. However, the potential advantages of a minimally invasive approach must be balanced against its main limitation: reduced operative exposure compared with full sternotomy. As previously emphasized by von Segesser et al., less invasive cardiac surgery involves a trade-off between a smaller incision and the safety and exposure provided by the conventional approach [12]. Therefore, careful patient selection, detailed preoperative planning, and readiness to convert to sternotomy if exposure or safety is compromised are essential [13].

## Case presentation

A 22-year-old woman with absent pulmonary valve syndrome diagnosed at birth underwent surgical correction during childhood in 2013, including implantation of a bioprosthetic pulmonary valve (Edwards N21) and pulmonary artery plication. Bioprosthetic valves are commonly used in cardiac surgery because of their lower risk of thrombosis and the avoidance of long-term anticoagulation therapy [1]. She remained clinically stable and symptom-free for 12 years after the initial operation.

Approximately one year before admission, she developed progressive exertional dyspnea associated with a decline in functional capacity, consistent with New York Heart Association functional class II [2]. Transthoracic echocardiography demonstrated findings suggestive of pulmonary bioprosthetic valve dysfunction; however, the available imaging did not allow accurate quantification of regurgitation severity or adequate assessment of pulmonary artery dimensions.

**Figure 1:**
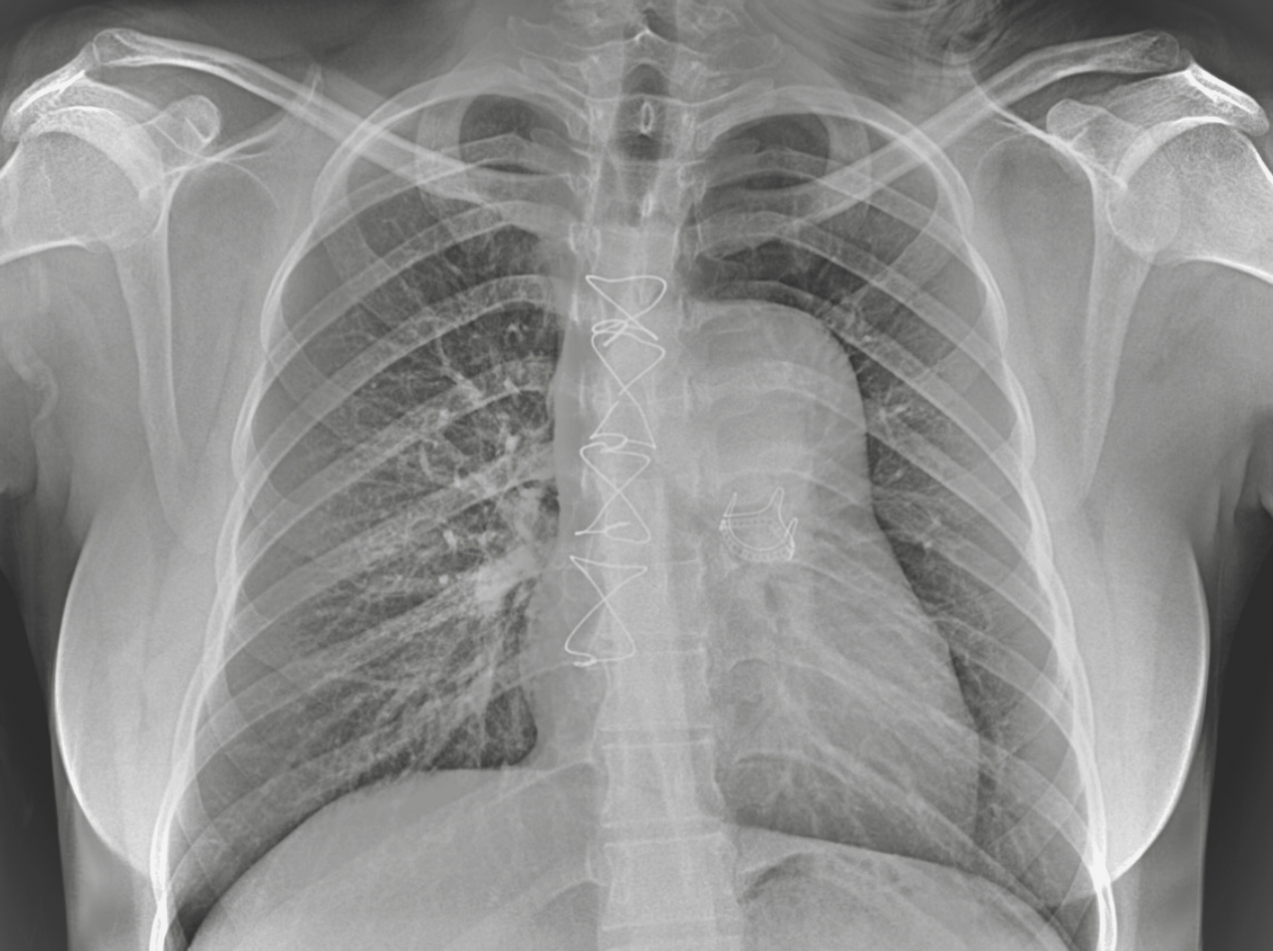
Preoperative X-ray. Chest radiograph obtained prior to surgical intervention showing an implanted 21 mm pulmonary bioprosthesis and aneurysmal dilation of the pulmonary trunk (~30 mm).

Preoperative chest radiography demonstrated pulmonary congestion and findings suggestive of right-sided volume overload, consistent with prosthetic valve dysfunction (Figure 1). Based on the clinical presentation and imaging findings, the patient was scheduled for reoperative pulmonary valve replacement.Preoperative femoral Doppler ultrasonography was performed to assess the size and patency of the groin vessels and to confirm the feasibility of peripheral femoral cannulation for cardiopulmonary bypass.

**Figure 2:**
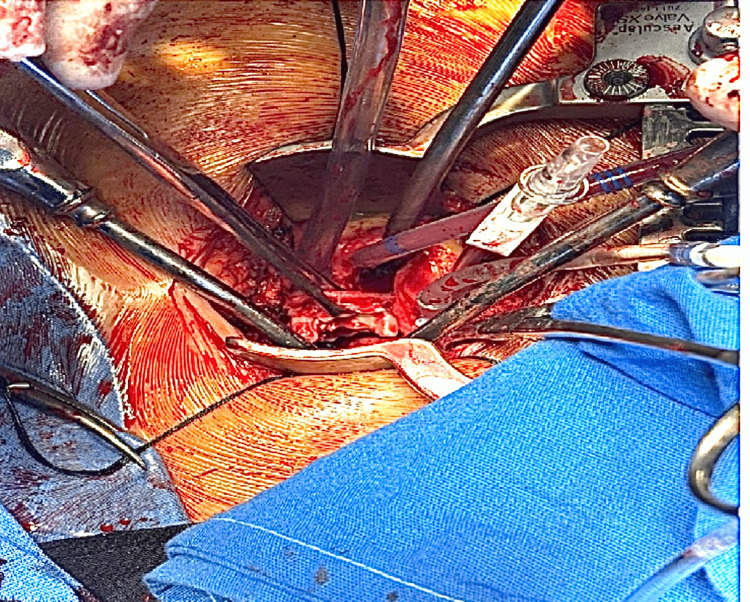
Intraoperative view during explantation of the degenerated pulmonary bioprosthetic valve through a left mini-thoracotomy approach. Intraoperative image showing removal of the degenerated pulmonary bioprosthetic valve during reoperative pulmonary valve replacement.

Surgical intervention was performed through a left mini-thoracotomy approach, allowing direct access to the right ventricular outflow tract and pulmonary artery (Figure 2).

**Figure 3:**
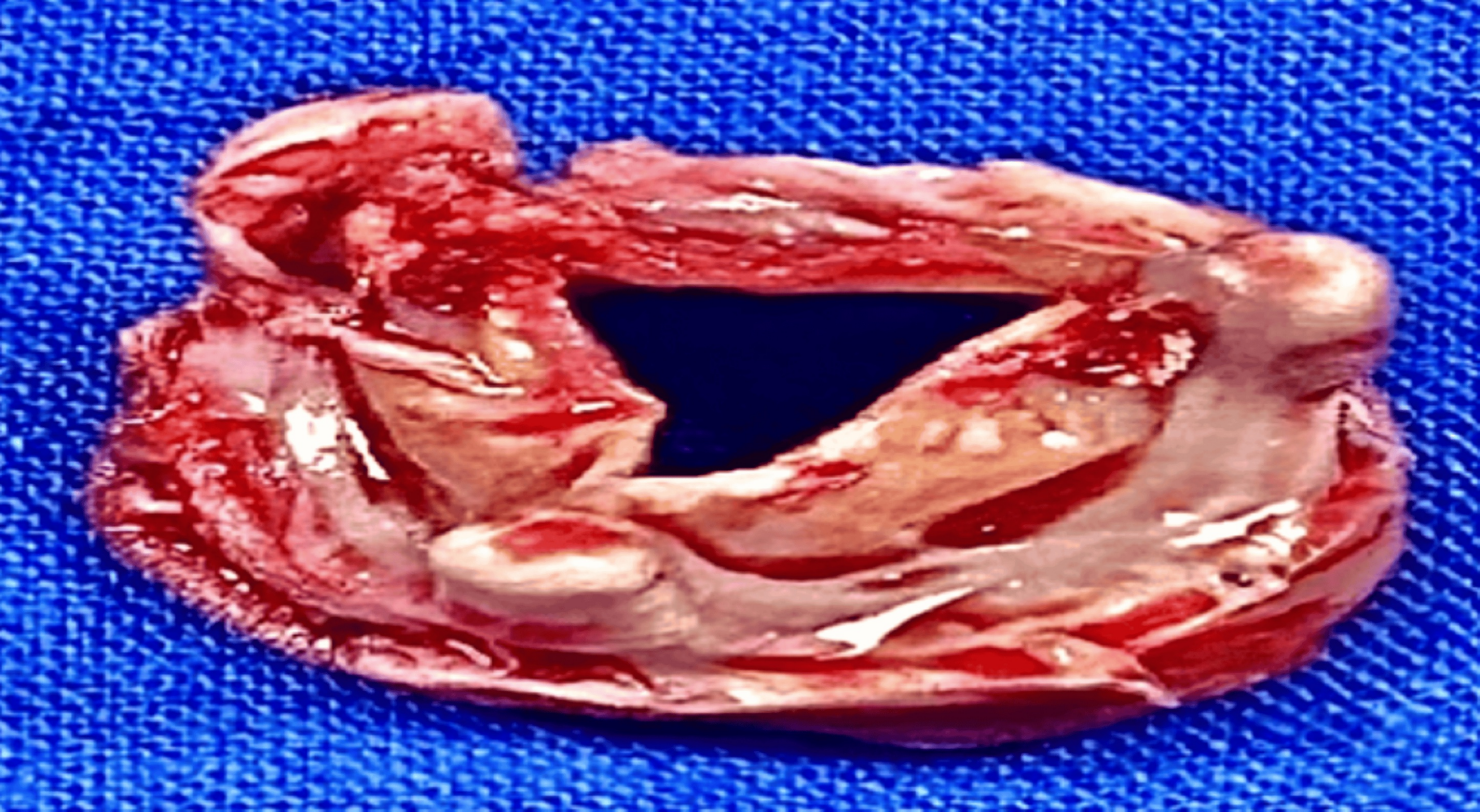
Explanted degenerated pulmonary bioprosthetic valve with structural deterioration and calcification. Gross specimen of the removed valve demonstrating advanced degeneration with calcification, consistent with prosthetic valve dysfunction.

Cardiopulmonary bypass time was 157 minutes, and aortic cross-clamp time was 92 minutes. Intraoperative findings included a dilated main pulmonary artery measuring approximately 30 mm in diameter and a severely degenerated, calcified pulmonary bioprosthesis with associated regurgitation (Figure 3). The previously implanted valve was explanted.

**Figure 4:**
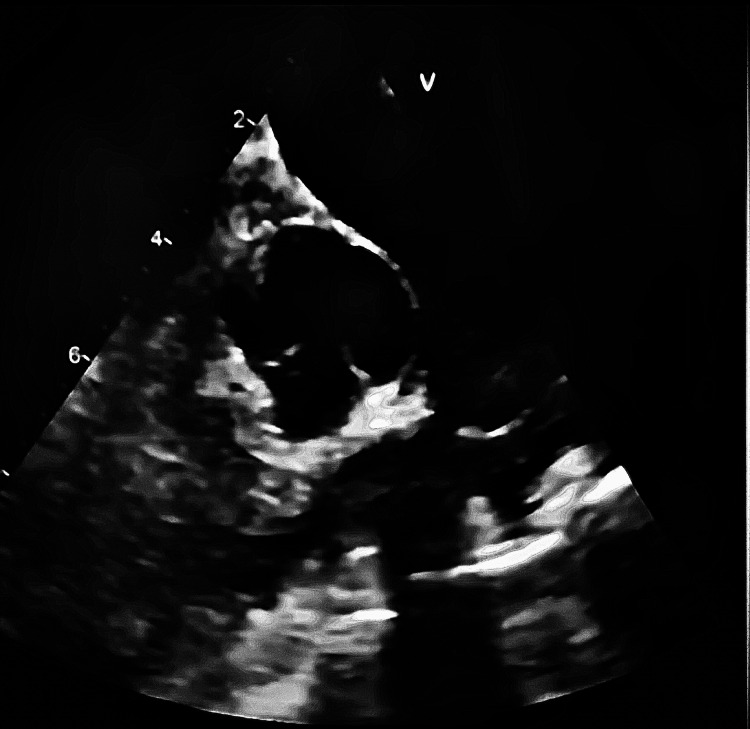
Intraoperative echocardiographic view of the pulmonary valve during surgical intervention. Intraoperative transesophageal echocardiographic image showing the prosthetic pulmonary valve region and right ventricular outflow tract. The image was optimized for resolution and labeling; however, quality reflects the inherent limitations of intraoperative acquisition.

**Figure 5:**
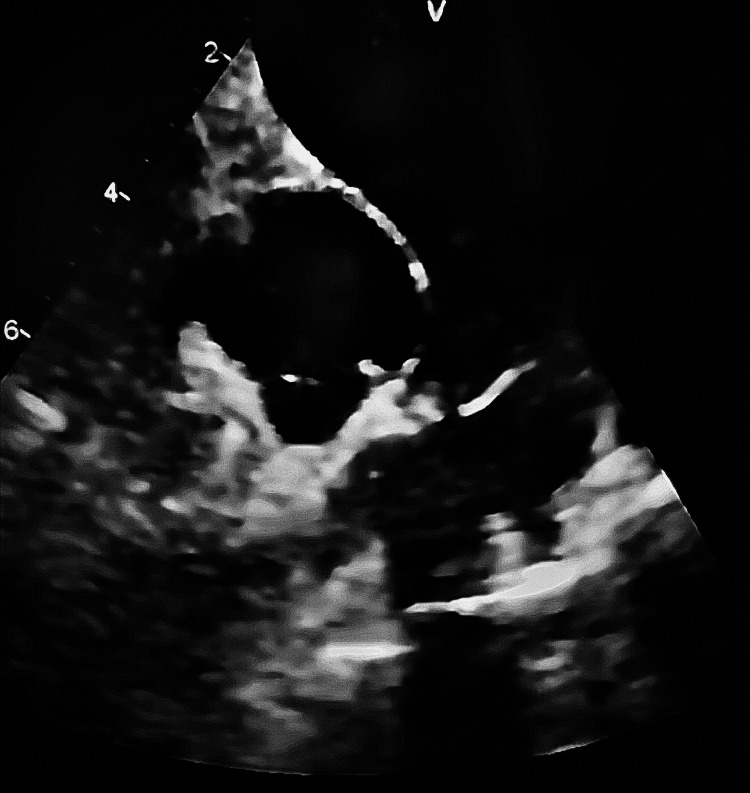
Postoperative echocardiographic view of the implanted pulmonary bioprosthetic valve. Postoperative echocardiographic image demonstrating the implanted pulmonary valve prosthesis with preserved structural appearance. This view allows anatomical assessment; however, detailed hemodynamic evaluation requires Doppler imaging.

A 25 mm bioprosthetic valve was implanted in the pulmonary position, restoring valve competence and improving right ventricular outflow tract dynamics. Intraoperative transesophageal echocardiography demonstrated adequate valve function, with no significant regurgitation or obstruction (Figures 4-5).

**Figure 6:**
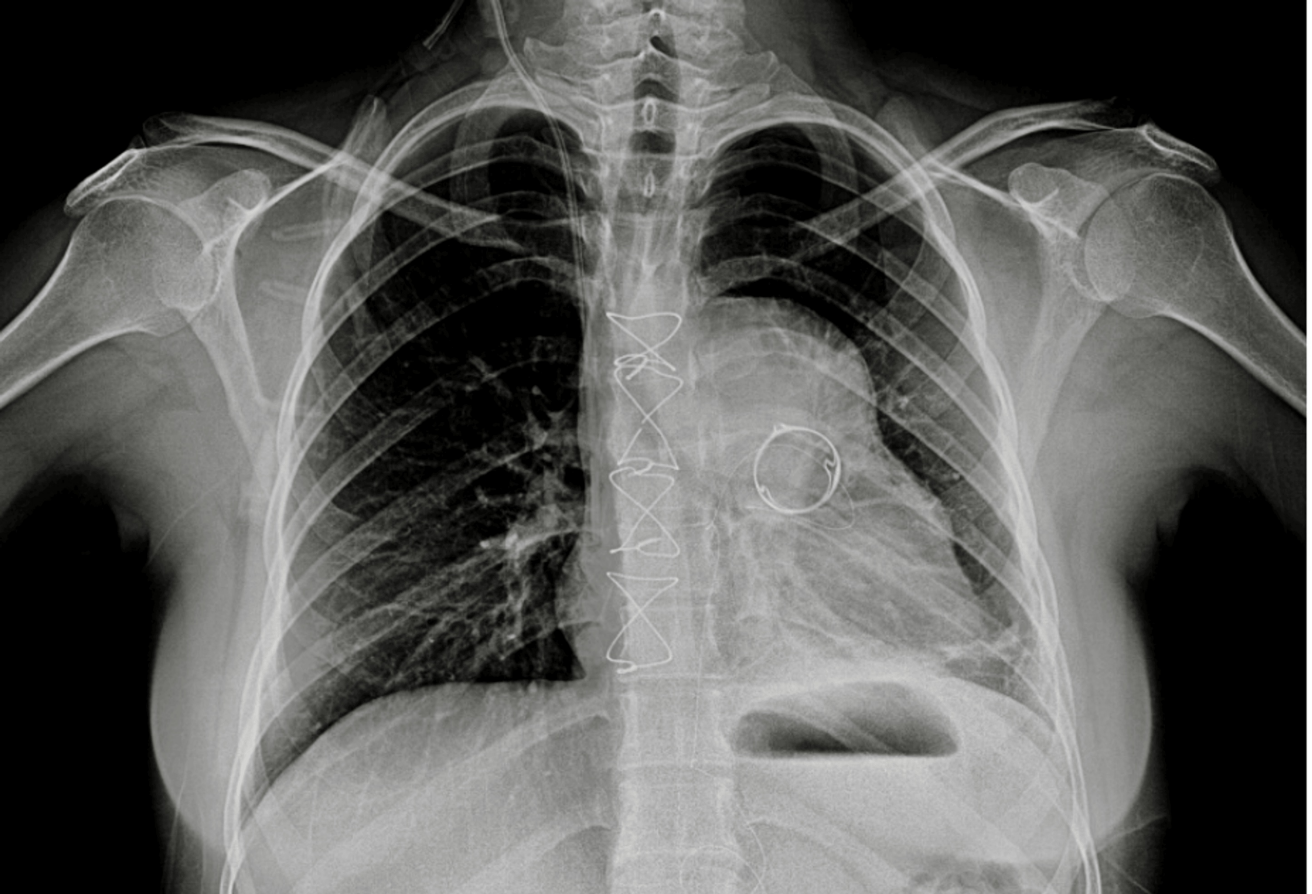
Postoperative chest radiograph demonstrating appropriate positioning of the pulmonary valve prosthesis, with improved pulmonary congestion compared to the preoperative study.

The postoperative course was uneventful. The patient was transferred to the postoperative care unit for monitoring and subsequently showed progressive clinical improvement. Follow-up chest radiography demonstrated resolution of pulmonary congestion and appropriate positioning of the newly implanted prosthesis (Figure 6). A limitation of this report is the absence of preoperative imaging adequately demonstrating the severity of pulmonary regurgitation and pulmonary artery dilation.

## Discussion

Pulmonary valve replacement is a well-established intervention in patients with congenital heart disease who develop progressive right ventricular outflow tract dysfunction, particularly in those with a history of absent pulmonary valve syndrome. Although early surgical correction improves survival, long-term outcomes remain limited by progressive right ventricular dilation and structural degeneration of bioprosthetic valves, often necessitating reintervention during adolescence or early adulthood [3,4]. In the setting of redo intervention, alternatives to repeat surgical pulmonary valve replacement should also be considered. Transcatheter pulmonary valve replacement (TPVR) has emerged as a less invasive option in selected patients, particularly those with favorable right ventricular outflow tract anatomy and dysfunctional prior conduits or bioprosthetic valves, offering the potential to avoid repeat sternotomy and reduce perioperative morbidity. However, its applicability remains dependent on anatomical suitability, prosthesis characteristics, and institutional expertise. Recent reports have further highlighted the feasibility of TPVR even in resource-limited settings, supporting its growing role as an alternative to conventional redo surgery in appropriately selected cases [5].

In the present case, the patient developed symptomatic deterioration more than a decade after initial repair, which is consistent with the expected lifespan of a bioprosthetic valve in the pulmonary position. Structural valve degeneration remains a predictable limitation, especially in younger patients, and represents one of the main indications for repeat pulmonary valve replacement [6].

Reoperative cardiac surgery after prior sternotomy is associated with increased technical complexity and perioperative risk due to mediastinal adhesions, scarring, and the potential for injury during re-entry. These risks may be particularly relevant in congenital heart disease patients who require repeat interventions after childhood repair, including those treated in low- and middle-income countries, where resource availability and access to specialized care may influence treatment strategies. Careful preoperative assessment, appropriate diagnostic imaging to define the operative plan, and cautious mediastinal dissection are essential to improve safety in redo cardiac surgery [7]. In this context, alternative surgical strategies have been developed to minimize these risks. Minimally invasive approaches, including left mini-thoracotomy, allow adequate exposure of the right ventricular outflow tract and pulmonary artery while avoiding repeat sternotomy in selected patients [8,9].

Recent studies have demonstrated the feasibility of pulmonary valve replacement through left thoracotomy and minimally invasive approaches, with favorable outcomes in selected patients undergoing reoperative procedures [9-15]. More recent reports, including case series and hybrid techniques, further support the reproducibility and safety of this approach in appropriately selected patients [13-15].

Although TPVR has emerged as an important alternative for selected patients with dysfunctional right ventricular outflow tract conduits or failed bioprosthetic valves, it was not considered the optimal strategy in our patient. She had a previously implanted 21 mm Edwards bioprosthesis, which in other settings could potentially serve as a landing zone for a valve-in-valve procedure. However, the presence of structural degeneration of the prosthesis, associated calcific changes, and dilatation of the main pulmonary artery made a purely percutaneous approach less favorable. In addition, surgical reintervention allowed direct visualization, complete explantation of the failed prosthesis, and definitive treatment through a less invasive left mini-thoracotomy. Institutional experience and local resource availability may also influence the choice of treatment strategy in this setting.

In this case, the use of a left mini-thoracotomy approach allowed safe explantation of the degenerated prosthesis and successful implantation of a new bioprosthetic valve, with an uneventful postoperative course and favorable early clinical outcome. These findings are consistent with the growing body of evidence supporting minimally invasive strategies in reoperative congenital cardiac surgery [9-15].

An important consideration is the durability of bioprosthetic valves in the pulmonary position. Although they offer significant advantages, including favorable hemodynamics and avoidance of long-term anticoagulation, their limited lifespan necessitates lifelong follow-up, as many patients will require subsequent interventions, either surgical or transcatheter [6].

## Conclusions

Pulmonary valve re-replacement remains a necessary surgical intervention in selected patients with congenital heart disease who develop structural degeneration of bioprosthetic valves. This case demonstrates that a left mini-thoracotomy is a feasible and safe surgical alternative in carefully selected patients undergoing reoperative pulmonary valve replacement after prior sternotomy.

This approach may provide adequate surgical exposure while avoiding the risks associated with repeat median sternotomy and may also facilitate postoperative recovery. Careful patient selection and surgical expertise remain essential to achieve favorable outcomes. However, longer-term follow-up and studies including larger patient cohorts are required to better define the durability, reproducibility, and comparative outcomes of this approach versus conventional repeat median sternotomy.
